# The Emerging Impact of Tumor Budding in Oral Squamous Cell Carcinoma: Main Issues and Clinical Relevance of a New Prognostic Marker

**DOI:** 10.3390/cancers14153571

**Published:** 2022-07-22

**Authors:** Lucrezia Togni, Vito Carlo Alberto Caponio, Nicoletta Zerman, Giuseppe Troiano, Khrystyna Zhurakivska, Lorenzo Lo Muzio, Andrea Balercia, Marco Mascitti, Andrea Santarelli

**Affiliations:** 1 Department of Clinical Specialistic and Dental Sciences, Marche Polytechnic University, Via Tronto 10/A, 60126 Ancona, Italy; l.togni@pm.univpm.it (L.T.); andrea.santarelli@staff.univpm.it (A.S.); 2 Department of Clinical and Experimental Medicine, University of Foggia, Via Rovelli 50, 71122 Foggia, Italy; vitocarlo.caponio@unifg.it (V.C.A.C.); giuseppe.troiano@unifg.it (G.T.); khrystyna.zhurakivska@unifg.it (K.Z.); lorenzo.lomuzio@unifg.it (L.L.M.); 3 Department of Surgery, Dentistry, Paediatrics and Gynaecology, University of Verona, Piazzale Ludovico Antonio Scuro 10, 37134 Verona, Italy; nicoletta.zerman@univr.it; 4 Department of Oral and Head-Neck Surgery, Umberto I General Hospital, Via Conca 71, 60126 Ancona, Italy; andrea.balercia@ospedaliriuniti.marche.it; 5 Dentistry Clinic, National Institute of Health and Science of Aging, IRCCS INRCA, Via Tronto 10/A, 60126 Ancona, Italy

**Keywords:** tumor budding, oral squamous cell carcinoma, tongue neoplasm, head and neck cancers, prognosis, lymph node metastasis, survival

## Abstract

**Simple Summary:**

The results of tumor budding are an independent prognostic factor of locoregional recurrences in many solid cancers, including colorectal, nasopharyngeal, esophageal, and lung cancer. Regarding oral squamous cell carcinoma, tumor budding predicts poor survival outcomes regardless of the tumor subsite and the pathological stage. Several detection methods for tumor budding have been proposed in the literature in order to reach an agreement on a standardized scoring system. However, the lack of a standardized assessment method prevents its evaluation in a multidisciplinary setting. In this context, this study aims to critically review the literature data regarding the prognostic role of tumor budding in oral squamous cell carcinoma. The validation of a tumor budding detection method based on tumor stage and site could be relevant to improving risk stratification and planning the clinical management of patients. Moreover, tumor budding could be included in daily pathological practice to better predict the patient’s outcomes.

**Abstract:**

Tumor Budding (TB) represents a single cancer cell or a small cluster of less than five cancer cells on the infiltrative tumor front. Accumulating evidence suggests TB is an independent prognostic factor in oral squamous cell carcinoma (OSCC). However, its exact role is not yet elucidated, and a standardized scoring system is still necessary. The study aims to extensively review the literature data regarding the prognostic role of TB in OSCC. The results of TB are an independent prognostic factor of poor survival outcomes in OSCC. To date, the manual detection of hematoxylin and eosin-staining or pancytokeratin-immunostaining sections are the most commonly used methods. Between the several cut-offs, the two-tier system with five buds/field cut-offs provides better risk stratification. The prognostic role of the BD model in predicting survival outcomes was extensively validated; however, the inclusion of DOI, which is already a staging parameter, encouraged other authors to propose other models, integrating TB count with other adverse risk factors, such as the tumor–stroma ratio and tumor-infiltrated lymphocytes. The prognostic relevance of TB in OSCC highlights its evaluation in daily pathological practice. Therefore, the TB detection method and the TB scoring system should be validated based on tumor stage and site.

## 1. Introduction

Tumor Budding (TB) represents a histopathological feature characterized by the presence of isolated single/small clusters of cancer cells dispersed within the stroma at the invasive tumor front. Gabbert et al. identified and described for the first time this histopathological feature at the infiltrative front of colorectal cancers. They defined budding as a “tumor dedifferentiation” due to several cytoarchitectural alterations such as a large nuclei, loss of desmosomes and junctional complexes, and a lesser degree of differentiation [1]. The term “budding” was proposed to highlight the fact that tumor cells and nests appeared to be budding out from larger tumor masses [2,3]. Subsequently, Ueno et al. defined TB as an isolated single tumor cell or cluster with less than five tumor cells located on the infiltrative tumor front [4].

**Figure 1:**
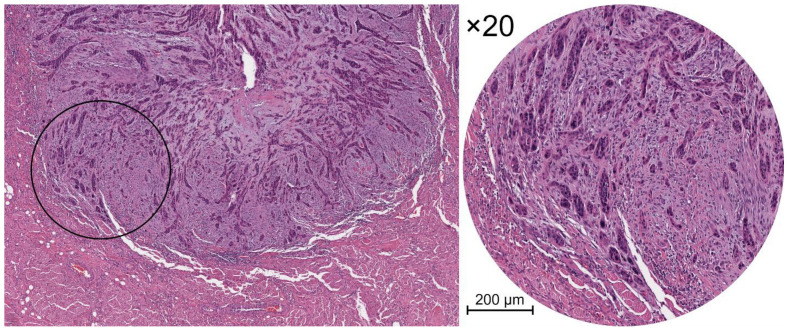
Histological views of tumor budding in H&E-stained sections of OSCC. **Left** panel: infiltrative tumor front at low magnification. **Right** panel: the inset shows a part of the same images at higher magnification (×20).

Several studies have reported TB as an independent prognostic factor of locoregional recurrences in many solid cancers, such as colorectal [5,6,7], nasopharyngeal [8], esophageal [9], pancreatic [10], and lung [11] cancers. In order to standardize the scoring system of this parameter, the International Tumor Budding Consensus Conference (ITBCC) for colorectal cancer proposed a method to assess TB [5]. The ITBCC recommends its evaluation using hematoxylin and eosin (H&E) stained sections using a cut-off of five buds/field, providing the total buds count to avoid any loss of prognostic information. To ensure the standardization of field size, the ITBCC group suggested an evaluation by an area equal to 0.785 mm^2^ (×20 objective lens with a 20 mm eyepiece field diameter), developing a conversion table for microscopes with different fields of view. Furthermore, the ITBCC group recommended the “hotspot” method, scanning 10 separate fields (×10) along the infiltrative tumor front and counting TB in the “hotspot” area (×20) (Figure 1) [6]. Based on these findings, Wang et al. proposed for the first time the evaluation of TB according to ITBCC recommendations in oral squamous cell carcinoma (OSCC) [5].

OSCC is the most frequent head and neck malignancy, with about 400.000 new cases diagnosed annually worldwide, accounting for over 90% of all oral cavity cancers [12,13]. The mortality rate is still high and has been stable for over two decades. In particular, the National Cancer Institute estimated about 54.000 new cases and 11.230 new deaths in 2022, with a short-term survival rate of about 65%. Despite advancements in the prognostic stratification of OSCC, the 8th edition of the American Joint Committee on Cancer (AJCC) staging system still needs to be improved to better identify those patients characterized by poor prognosis. This is especially true in light of the lack of new validated prognostic markers for the risk stratification of OSCC patients [14].

Currently, researchers have been focusing on the histological characteristics of the tumor microenvironment (TME) as a source of reliable markers for cancer progression. Accumulating evidence suggests TB as an independent prognostic factor in OSCC [15,16,17,18,19,20,21,22,23,24,25,26,27] and in oral tongue squamous cell carcinoma (OTSCC), the most involved oral subsite [22,23,26,28,29,30,31,32,33,34,35,36,37,38,39,40,41]. Nevertheless, due to several methodological shortcomings, the exact role of TB in OSCC is not yet elucidated, and conflicting results have been reported. Therefore, the aim of this narrative review is to overview and summarize the evidence from the literature on the clinical and prognostic role of TB in OSCC.

## 2. Tumor Budding in Oral Squamous Cell Carcinoma

### 2.1. Tumor Budding and the Epithelial-Mesenchymal Transition

TB seems to develop a dynamic phenotype that characterizes aggressive and invasive tumors. Indeed, neoplastic epithelial cells tend to lose cellular adhesion to resist apoptosis and increase peritumoral stroma degradation and their invasion ability [15,17,25,32,40,42,43,44,45]. The infiltrative tumor front is essential for cancer progression, representing the area of active invasion and cross-talking between tumor and stroma. Therefore, TB could represent an epithelial cell nest undergoing epithelial–mesenchymal transition (EMT) or dedifferentiation [15,17,25,29,32,39,43,44,45,46,47]. EMT usually occurs during embryogenesis, when polarized epithelial cells undergo a mesenchymal transition, enhancing their activation, migration, and proliferation and the production of the extracellular matrix [17]. However, once differentiated, these cells can undergo the reverse process, called mesenchymal–epithelial transition (MET). The EMT process is also a hallmark of several human cancers since it is involved in cellular reorganization and in extracellular matrix remodeling to promote neoplastic dissemination. The differential expression of several crucial EMT-related genes in oral cancer cells, such as E-cadherin, β-catenin [10,17,40,43,46], Vimentin [17,40,48], Claudins [49], Laminin 5-γ2 [30,50,51], N-cadherin, α-smooth muscle actin, and Fibronectin [17,38,52] support the EMT process of bud cells. In OTSCCs, high TB has been associated with a downregulation of E-cadherin and the upregulation of Vimentin expression [40]. Similarly, the upregulation of Vimentin and β-catenin and a loss of E-cadherin expression in the tumor buds of early-OSCCs have been demonstrated [43]. Nuclear β-catenin localization and the subsequent activation of Vimentin could provide the biological rationale for the gain of a mesenchymal phenotype [43]. However, the retention of N-cadherin and the occasional up-regulation of Vimentin suggested only a partial EMT in early-OSCCs [17,43]. The overexpression of transforming growth factor β (TGF-β) and EMT-transcriptional factors (EMT-TFs), such as Zinc finger E-box binding homeobox 1 (ZEB1), Fibronectin 1, and Collagen Type 1 and Type 5, and the downregulation of MET-TFs, such as Ovo-Like Transcriptional Repressor 1 (OVOL1), in the neoplastic cells of the infiltrative front rather than the adjacent stroma, corroborated the hypothesis of a partial EMT of bud cells [17,43], as well as the downregulation of miR-200 and miR-141 of cell buds from the neoplastic central area to the infiltrative tumor front [17].

### 2.2. The Evaluation of Tumor Budding in Oral Squamous Cell Carcinoma

#### 2.2.1. Manual vs. Digital Detection

To date, two methods of TB assessment have been used in OSCC: manual identification and digital detection, both of which can be performed on H&E-stained slides or on pan-cytokeratin-immunostained sections (Table 1). H&E manual evaluation is the most commonly reported method in the literature, and several meta-analyses confirm that the prognostic role of TB does not change between conventional and immunohistochemical (IHC) evaluation. However, the peritumoral infiltrate, cancer-associated fibroblasts, and the other reactive stromal cells, as well as glandular fragmentation, could prevent its identification [26,29,40,53]. Moreover, the isolated bud cells are usually inconspicuous in H&E slides because the borders of epithelial cells can be easily confused with the adjacent stroma [45,54]. On the contrary, pancytokeratin immunostaining better defines the limits of bud cells [53,54], simplifying its identification when the peritumoral infiltrate is abundant [26,29,40,53]. Nevertheless, isolated cells with multiple nuclei and spindle cells that are not distinguishable from fibroblasts and/or endothelial cells, as well as apoptotic bodies and cellular debris, should not be counted [40]. In OSCC, a shorter working time and better reproducibility for IHC evaluation were reported, especially for junior examiners [45,53,54,55]. Indeed, the intraobserver agreement of IHC was almost perfect for junior, senior, and expert examiners, while on H&E slides, substantial and moderate agreements were found for junior, senior, and expert examiners, respectively. The concordance analysis between the two methods showed a fair agreement for junior examiners and a moderate agreement for seniors and experts [54,55,56,57]. The IHC evaluation was easier in comparison to H&E staining, regardless of the examiners’ experience [54,55]. Finally, due to the characteristics of the method, the IHC assessment identified a greater number of OSCC cases with a high TB value (≥5 buds/field) [55].

A quantitative and semi-automatic digital image analysis algorithm, called “Digital Tumor Bud Count”, has been proposed by Pedersen et al. All of the analyses are performed by the software (Visiopharm A/S, Hoersholm, Denmark), except for the manual selection of the hotspot area on IHC sections (×20) to exclude the salivary glands and necrotic areas that could be not recognized by the software. The image analysis is based on the brown color thresholding emitted by the chromogen-stained areas to discriminate them from the unstained areas. According to this algorithm, the TB value resulted in being the most powerful prognostic factor for overall survival (OS) and lymph node metastasis (LNM) in early-OSCCs, both as a quantitative and continuous variable. Notably, its prognostic value linearly decreased as the tumor island area increased. Thus, a cut-off point of the evaluation area equal to 950 μm^2^, corresponding to clusters composed of less than five cells, has been selected. The Digital Tumor Bud Count algorithm showed better accuracy and reproducibility compared to the manual method, so that it could overcome the major limitations of TB detection [58].

**Table 1:** Parameters and modalities of TB evaluation in Oral Squamous Cell Carcinoma explain in literature.

Parameter	Modalities Reported in Literature [References]
TB counting approaches	Manual detection [15,18,19,24,25,26,28,29,30,31,32,33,34,36,37,38,40,43,53,54,55,56,57,59,60,61,62,63,64,65,66,67,68,69] Digital detection [58] Semiautomated detection [17,20]
Cut-off values	No cut-off [17,20,22] Dichotomic cut-off (presence/absence) [25,70] Single cut-off: - 3 buds/field [31,32,44,64] - 4 buds/field [55] - 5 buds/fields [16,23,24,26,29,33,34,37,38,40,43,53,58,59,60,63,64,67,69,71] - 10 buds/field [15,19,61,62] - 15 buds/field [24] Double cut-off: - 0 and 5 buds/field [68,72] - 3 and 5 buds/field [18] - 5 and 10 buds/field [28,29,36,64,65,73]
Counting parameters	n. of HPF evaluated: 1, 5, 10 - 1 [5,15,16,18,19,23,24,25,26,28,29,30,32,34,36,37,38,40,43,44,53,54,55,56,57,60,61,62,63,64,65,67,68,69,71,73,74] - 5 [15,33] - 10 [19,24,66] n. of TB reported: - per HPF [17,20,21] - per mm^2^ [5,20,28,29,58] Magnification - ×10 [62] - ×20 [16,17,18,23,25,26,28,29,30,32,34,36,37,38,40,43,44,53,54,55,57,58,59,60,61,63,64,68,69,70,71,73,74] - ×25 [15] - ×40 [24,25,31,66]
Staining technique	H&E [15,16,23,24,25,26,28,29,31,33,34,38,40,44,53,54,57,60,62,63,64,67,68,69,70,74] IHC [18,19,20,22,30,32,37,38,43,53,54,55,56,57,59,65,71,73]

TB: Tumor Budding; H&E: haematoxylin and eosin; IHC: Immunohistochemistry; HPF: High power field.

#### 2.2.2. Preoperative Biopsy vs. Postoperative Surgical Specimen

Few studies have evaluated TB on incisional biopsies [18,30,32,56,57,59,60,61,71]. Several technical limitations hinder its accurate assessment, such as the small sample size, the lack of the infiltrative front, sample fragmentations, artifacts, and extensive necrosis. However, some authors have highlighted the potential usefulness of a preoperative evaluation of TB in representative biopsies, including the deepest part of the primary tumor [32,60,62]. Moreover, preoperative TB showed strong correlations with grading, the depth of invasion (DOI), lymph vascular invasion (LVI) [75], and the pattern of invasion (POI) [71]. Therefore, it has been suggested to perform a biopsy that includes clinically healthy tissue with a horizontal margin of p ≥ 8 mm and a vertical margin of ≥5 mm [59] or perform several incisional biopsies [60]. In colorectal cancer, it has been recommended to perform at least three biopsies [76]; thus, this procedure might also be useful in oral cancer. Preoperative TB results in being a good predictor of DFS and LNM in early-OSCC [18] and demonstrates an excellent specificity (100%) in predicting the postoperative values of OTSCCs [60]. However, a recent study demonstrated the low specificity of preoperative TB values in predicting Extranodal extension (ENE), despite the fact that it showed a significant association with the number of metastatic lymph nodes [61]. In OSCC, the evaluation of the small intratumoral cell nest size (CNS) (<5 buds/field) and the budding activity (BA) on fresh frozen sections results in a prognostic factor of patient outcomes [24].

### 2.3. Tumor Budding Scores in Oral Squamous Cell Carcinoma

To provide a risk stratification for OSCC patients, different TB cut-offs have been suggested. The five buds/field cut-off is the most commonly used [26,30,38,40,43,50,53,54,56,57,59,60,63,64], followed by three buds/field [23,31,32,44,64], four buds/field [55], and ten buds/field [19,28,29,62,65,73] cut-offs, while only two studies adopted a dichotomic approach (presence/absence of tumor buds) [25,70] (Table 1). For OSCC risk stratification, most studies have used a two-tier system with a TB cut-off equal to five buds/field. However, to improve its predictive value, the ITBCC recommends the three-tier system of the Japanese Society for Cancer of the Colon and Rectum [5]. This system classifies patients into low, intermediate, and high TB cases (≤4, 5–9, and ≥10 buds/field, respectively) and has been verified both on OTSCC [29,64] and OSCC [65,73]. No significant differences in survival outcomes were shown between intermediate and high TB cases [29,64]. Moreover, only high TB was correlated to LNM and DSS [65], suggesting ITBCC cut-offs might not clearly stratify OTSCC patients’ outcomes. Despite the fact that an evaluation based on categorical data is more practical in clinical trials, TB is per se a continuous variable; thus, a continuous scale evaluation could provide a more accurate risk stratification.

### 2.4. Tumor Budding Risk Models in Oral Squamous Cell Carcinoma

The first risk model based on TB was the BD model, proposed by Almangush et al. The BD model is a three-tier system (BD0, BD1, and BD2) that evaluates the H&E-stained sections of both TB and DOI in order to predict loco-regional recurrence and OS in early-OTSCC cases (Table 2) [38]. 

Since upper and lower aerodigestive carcinomas are closely related, Boxberg et al. proposed a risk model that is already validated in esophagus squamous cell carcinoma to evaluate the risk stratification of OSCCs on H&E-stained sections. Based on the BA score (BA1, BA2, and BA3) and intratumoral CNS score (CNS1, CNS2, CNS3, and CNS4), a combined grading system for OSCCs has been proposed: well-differentiated tumors with a score equal to 2–3 (G1); moderately-differentiated tumors with a score ranging from 4 to 6 (G2); and poorly-differentiated tumors with a score equal to 7 (G3) (Table 2) [66].

Another prognostic model, the iBD model, has been proposed for OTSCC-risk stratification [67]. By matching the Glasgow Microenvironment Score [77] and BD score [38], Yu et al. classified OTSCC into three different risk groups (iBD0, iBD1, and iBD2) (Table 2). Elseragy et al. incorporated the H&E evaluation of TB and the WHO grading system into the revised grading (RG) system to improve its prognostic value in early OTSCCs, by stratifying tumors into three risk groups: RG1, RG2, and RG3 (Table 2) [68].

Further, TB was integrated into the tumor–stroma ratio (TSR), which represents the ratio between malignant epithelial cells and the tumor-associated stroma in neoplastic tissue, to build a three-tier system for OSCC classification (low-risk, intermediate-risk, and high-risk tumors) (Table 2) [16].

Another approach using a semiautomated analysis of TSR and TB, combined with an IHC evaluation of tumor infiltrated lymphocytes (TILs), has been used to stratify OSCC patients. Based on six values (TSR, TB/tumor bed, TIL bed/tumor bed, CD8/stroma, CD3/stroma, and CD8/CD3), a total score ranging from 0 to 6 is determined by assigning one point to each unfavorable variable. Following the total score, OSCC cases are further classified into three risk groups as follows: score 0–3 (low-risk group); score 4–5 (intermediate-risk group); and score 6 (high-risk group) (Table 2) [20].

Finally, Hori et al. proposed an additional stratification factor for early-OTSCCs based on the total number of tumor CD163+ macrophages that are counted by a computerized image system and the BD model. The authors created a three-tier system for OSCC classification (Group 1, Group2, and Group 3) (Table 2) [36].

### 2.5. Prognostic Role of Tumor Budding in Oral Cancer

Despite the improvements to prognostic stratification in the 8th edition of the AJCC staging system, it fails to identify oral cancers that are characterized by poor prognosis [14]. Therefore, clinicians would need a predictive model that represents a personalizable therapeutic guide. The analysis of the morphological features of TME demonstrated the prognostic value of TB that significantly correlated with a high incidence of locoregional recurrences in many solid cancers (Table 3) [5,6,7,9,10,11]. In OSCC, a recent meta-analyses showed that TB is significantly correlated with OS [22,42,45,78], DSS, and DFS [78], regardless the pathological stage. Similarly, TB resulted in being an independent predictor of LNM [35,36,42,61], DSS [64], and OS [35,61] in OTSCC.

It is noteworthy that, according to the available data in the literature, OTSCC is characterized by different molecular and pathological features with respect to oral cancer of other subsites, hindering both research and clinical decision-making [72,79,80]. Therefore, a separate chapter has been reserved for discussing the prognostic role of TB in OTSCC.

The first study evaluating the prognostic role of TB in OSCC was conducted by Angadi et al., showing an association between high TB and LNM [15]. These findings were confirmed by several authors, both considering the total buds count [17,19,21,25], the three buds/×20 cut-offs [44], and the ITBCC system [28]. Moreover, TB significantly predicted a poor OS and a worse DFS in advanced and early OSCC patients [20], regardless of the oral subsite of the tumor (tongue vs floor of mouth) [17]. Only one study did not confirm the role of TB in predicting the LNM in gingiva–buccal complex carcinomas, highlighting the fact that only the worst POI (WPOI) resulted in being an independent predictor of survival outcomes. It could be caused by the presence of TB itself, which can affect the evaluation of WPOI. Moreover, the authors used a 10-buds/field cut-off, grouping patients with 5–9 buds/field in the low-risk group [73].

Furthermore, TB showed a significant correlation with positive surgical margins [16,17], younger age, and oral tongue and floor-of-the-mouth subsites [17].

With respect to the elderly, the immune system of young patients secretes more chemokines, cytokines, and growth factors, promoting the EMT process and supporting TB formation. Moreover, in young patients, the OTSCC represents the most common clinical form of oral cancer. The lymphovascular network and the muscular structure of the oral tongue and the floor of the mouth may contribute to tumor spread and EMT. A significant association also occurred with advanced pathological stage, larger tumor size, deeper invasion, infiltrative POI, LVI, and PNI [15,21,25,28,67]. Hypoxia and inflammation represent the major mechanisms promoting EMT. During tumor growth, hypoxia could directly influence the behavior of cancer cells by triggering EMT, promoting the neoplastic dissemination and the formation of bud cells. Deeper invasion and infiltrative POI may contribute to the host immune and inflammatory reaction, further promoting the EMT [25]. It has also been reported that high TB is an excellent predictor of ENE (+), especially in combination with DOI evaluation. High TB value in OSCCs showed a 3.3-time higher risk of ENE (+) compared to low TB tumors [28]. Finally, in OSCCs with fat or muscular invasion, the presence of TB had a four-times increased risk of locoregional recurrence compared to tumors without TB [74]. Furthermore, the role of cytoplasmic pseudofragments, defined as no-nucleated TB areas at the infiltrative tumor front with a 2 μm diameter, was investigated. Their presence was associated with high TB [53] and pN (+) OSCCs [20]. Regarding early OSCC, TB represented the only significant prognostic factor of locoregional recurrences, showing a 3.5 times higher risk in high-TB tumors compared to low-TB tumors [31]. Furthermore, high TB resulted in being an independent predictor of occulted LNM [18,25,73] and worse DFS and OS [18,73], especially in the oral tongue and gingiva subsites [18]. Significant correlations were also observed between TB and grading, DOI [18], LVI [18,73], PNI [73], and POI [18,31,73]. However, the association has not been found for WPOI-5 [73]. The WPOI-5 evaluation is characterized by small tumor islands located at least 1 mm away from the main tumor mass, and it has been recognized as significantly predictive of worse outcomes. Since TB assessment depends on the number of tumor cells in a single tumor island, the prognostic role of WPOI and TB in early OSCC may be conditioned to their different definition criteria [73].

#### Prognostic Role of Tumor Budding Risk Model in Oral Squamous Cell Carcinoma

The prognostic role of the BD model in predicting DSS was also validated in OSCC [69]. However, the inclusion of DOI represents a major criticism of the BD model because DOI is already a staging parameter [24]. In particular, Boxberg et al. found no significant difference between BD0 and BD1 groups, suggesting an inadequate stratification between low- and intermediate-risk patients [24]. 

The model proposed by Boxberg et al. showed a significant association between combined grading and the risk of LNM. According to this model, G3-OSCCs are more frequently diagnosed in advanced pathological stages compared to G1/G2-OSCCs. Moreover, CNS and BA scores resulted in being prognostic factors of OS, DSS, and DFS. The evaluation of combined grading both on early (pT1/2) and advanced (pT3/4) OSCCs subgroups and on the nodal negative (pN0) and positive (pN1/2) subgroups separately, confirmed its prognostic value. The same researchers obtained excellent interobserver and intraobserver variability both for BA, CNS, and combined grading in a larger cohort of OSCC [66].

Regarding the iBD model, strong correlations between TB and inflammatory reaction and TSR were identified: a higher TB was associated with both lower inflammatory reaction and higher TSR. Furthermore, BD score and inflammatory reaction resulted in being negative predictors of poor OS and poor DFS in OSCC, suggesting the critical role of stromal reaction and local inflammation in TB development. Therefore, A dicreased local antitumoral activity and a higher TSR could promote the differentiation and dissemination of neoplastic cells along the infiltrative tumor front [67]. However, when comparing the BD and iBD models, the BD model resulted in better predictions of worse DSS, especially in early-OSCC [69]. 

Finally, the risk model proposed by Duorato et al. resulted in being an independent prognostic factor for DSS and DFS, both in OSCC and early-OSCC [16]. Similarly, the model proposed by Sung et al. significantly stratified OSCC patients characterized by poor prognosis [20]. The importance of inflammation and tumor stroma emerges from the results obtained by the different research groups, suggesting that the inclusion of TSR and inflammatory reaction into a risk model should be further investigated. 

### 2.6. Prognostic Role of Tumor Budding in Oral Tongue Squamous Cell Carcinoma

Wang et al. investigated for the first time the role of TB in OTSCC. These authors showed the independent prognostic role of high TB in predicting LNM. Later, these data were corroborated by several studies [22,29,30,31,32,34,36,37,61,65,67]. Notably, occulted LNM were found in all OTSCCs with ≥3 buds/×20 [31,32], suggesting a prophylactic supraomohyoid neck dissection for these cases [32]. Moreover, TB was able to predict DSS, both using a two-tier system (five buds/×20) and a three-tier system (ITBCC system) [64,67]. Finally, TB resulted significantly correlated whit DOI [64], POI [26,28,63], PNI [64], tumor size, grading, and pathological stage [40,64].

#### Prognostic Role of Tumor Budding Risk Model in Oral Tongue Squamous Cell Carcinoma

The validation of the ITBCC recommendations for OTSCC samples showed that the Bd score was significantly associated with LNM, local recurrences, grading, lymphoid infiltration, infiltrative POI, and DOI. Intermediate and high-risk groups exhibited lower OS and DSS compared to low-risk OTSCCs, also in early-OTSCCs, without significant survival difference between Bd2 and Bd3 groups, suggesting that the ITBCC cut-offs might not clearly stratify OTSCC patients [29,64]. Hence, it is always recommended to report the total TB count for each case to avoid any loss of prognostic information. Even the BD model resulted in being an independent predictor of local recurrences and OS in intermediate and high-risk groups compared to low-risk tumors [29,38,63]. In early-OTSCC, the BD model showed a predictive prognostic value for occulted LNM [23,26,28,37], DSS [34], and OS [28,38,63]. Notably, the combination of DOI ≥ 3 mm and high TB better predicts LNM, displaying a good sensitivity (89%) and excellent specificity (95%) [23,37]. Moreover, a DOI of 3.3 mm and a TB of four buds/field resulted in more accurate cut-offs for predicting occulted LNM [55]. However, the inclusion of DOI in the BD model has been questioned since it is already a staging parameter [24], and the prognostic role of the BD model has not been demonstrated by recent data [24,64]. Moreover, the risk model proposed by Hori et al. was able to predict the occulted LNM in group 3 compared to groups 1 and 2 [36]. Revised Grading was able to predict a worse DSS and DFS in RG3 rather than in the G3 groups, and an upgrading of 116 early-OTSCCs was demonstrated using the RG compared to WHO grading [68]. However, no significant differences in DSS and DFS were detected between the risk groups [64]. Recently, the ITBCC model showed a better prognostic stratification capability (0.739), followed by the BD model (0.735) and the five buds cut-off (0.735) [64].

### 2.7. Clinical Relevance of Tumor Budding in the Therapeutical Management of Oral Squamous Cell Carcinoma

The management of patients affected by oral cancer is mainly based on pathological staging, although it seems to be more accurate in the prognostication of advanced OSCC rather than early-stage tumors. This emphasizes the critical need for reliable prognostic factors to better define the risk stratification and provide clinical guides for personalized therapy. In 2016, the National Comprehensive Cancer Network (NCCN) Clinical Practice Guidelines recommended the surgical resection of primary cN0-OSCC, with or without ipsilateral neck dissection, depending on tumor thickness or bilateral neck dissection, based on the primary tumor site. Alternatively, surgical resection with or without sentinel lymph node biopsy or definitive radiation therapy was recommended. The complexity of the lymphatic circulation of the head and neck region makes the sentinel lymph node biopsy a difficult and time-consuming procedure. Moreover, this approach does not show an excellent accuracy in predicting LNM in head and neck cancers. In the presence of more than one adverse risk factor, such as ENE (+), PNI, LVI, positive resection margins, advanced pT, and pN (+), the NCCN Guidelines recommend an adjuvant systemic therapy or radiation therapy. Advanced-OSCCs are treated by surgical resection and ipsilateral or bilateral neck dissection, followed by multimodality therapy (adjuvant systemic therapy or radiation therapy), depending on risk factors. A trend of lower relapses has been observed in tumors with high TB treated by surgery and postoperative radiation therapy compared to surgery only. This could indicate a radio-sensibility of bud cells, suggesting that high-risk patients could benefit from adjuvant therapy. However, low-risk tumors recurred despite postoperative radiation therapy, probably due to the radio-resistance of EMT cells or cancer stem cells [43]. Neo-adjuvant chemotherapy clearly affected TB score. No-chemotherapy patients showed a significantly higher TB score rather than chemotherapy ones. However, between the 7th and 21st day of chemotherapy, some OSCCs showed an increase in TB score with the presence of bud cells scattered and embedded within the fibrous tissue. Instead, the TB score gradually decreased after the third week of chemotherapy, suggesting that the apparent increase in tumor buds in the first weeks could be defined as “pseudo-TB”. Indeed, as chemotherapy leads to cell adhesion disruption and cancer cell degeneration, is it possible that TB may be temporarily increased because of tumor degradation itself [59]. An elective neck dissection (END) is recommended for tumors with a DOI ≥ 4 mm; however, if DOI is equal to 2–4 mm, it can be performed according to clinical judgment. LNMs were detected in 5.6% and 16.9% of tumors with a DOI equal to 3 mm and 4 mm, respectively [81]. Thus, the indications for END only based on DOI could result in overtreatment. Although DOI has been lately added to the pT category and WPOI was suggested as an additional adverse risk factor in the 8th Edition of the AJCC classification, TB could represent a better predictor of occulted LNM. In early-OSCCs, risk group stratification could be relevant to selecting those patients that could undegro END and/or adjuvant radiation therapy. During the surgical treatment of early-OTSCCs, Almangush et al. recommend performing a pancytokeratin staining of frozen sections to identify high-risk tumors that could benefit from END and carry out the END without interrupting the anaesthesia [38]. To identify LNMs in early-OSCCs, Shimizu et al. proposed evaluating both the TB and the POI on biopsies and surgical specimens by using pancytokeratin staining and identifying those areas with >5 buds and POI-4C/4D patterns. If these features are present, the authors suggested a short-term follow-up (e.g., every one or two weeks for 6 months) with or without imaging. In case of clinical signs of nodal relapse, neck dissection should be conducted as soon as possible, but if >10 buds and the POI-4D or WPOI-5 patterns are observed in the same section, a wide resection with END should be performed [73].

## 3. Conclusions

Growing evidence shows that TB seems to reflect the biological aggressiveness of cancer cells, representing a phenotype undergoing partial EMT and promoting tumor invasion and metastasis. The evaluation of TB could enhance the pathologic staging and grading of oral cancer to better predict the patient’s outcomes. The identification of TB in OSCC is straightforward since pathologists can easily identify the number of tumor buds on H&E-stained sections. Furthermore, simplicity, reproducibility, and low cost of H&E staining is a factor that allows a worldwide evaluation of TB. These aspects make the implementation of TB in clinical practice as a new prognostic marker for OSCC particularly attractive. However, several methodological inconsistencies are reported, in particular: (1) only retrospective observational studies have been conducted until now, mainly based on a single-institution experience; hence, the presence of selection bias cannot be avoided; (2) cut-off values for tumor buds in OSCC patients vary considerably, (Table 2), and the most reliable value for defining the prognostic role of TB is still a subject of debate; (3) other parameters reported in the literature (i.e., manual or digital detection for TB counts, H&E or other staining techniques, and the magnification used) might influence the number of tumor buds detected. Finally, the excessive fragmentation of the specimens and the presence of extensive inflammation or necrosis can prevent a proper assessment of TB. Despite such shortcomings, numerous studies agree that a high number of tumor buds is significantly associated with DFS, OS, DSS, and LNM in OSCC (Table 3). Therefore, the prognostic relevance and high reproducibility of TB in oral cancer emphasize its inclusion in daily practice. Additional retrospective multicentre studies and prospective trials, as well as accuracy analysis, are required to standardize TB assessment methods as well as to establish an accurate TB score depending on the oral subsite. Notably, the prognostic value of TB in gingivae, cheek, and sinuses where the DOI is not suitable to predict the tumor behavior, should be analyzed. Therefore, TB evaluation may provide new clinical guides to select high-risk patients with occulted LNM to lead therapeutic management.

## Figures and Tables

**Table 2:** Tumor Budding risk models in Oral Squamous Cell Carcinoma.

Author, Year (Model Name)	Parameters	Cut-off	References
Almangush et al., 2015 (BD Model)	TB DOI	**BD0**: TB < 5/×20 and DOI < 4 mm **BD1**: TB < 5/×20 and DOI ≥ 4 mm or TB ≥ 5/×20 and DOI < 4 mm **BD2**: TB ≥ 5/×20 and DOI ≥ 4 mm	[24,33,34,38,64,69]
Lugli et al., 2017 (ITBCC Model)	TB	**Bd1**: TB ≤ 4/0.785 mm^2^ **Bd2**: TB 5–9/0.785 mm^2^ **Bd3**: TB ≥ 10/0.785 mm^2^	[5,28,29,34,65,73]
Boxberg et al., 2017	BA CNS	**G1**: 2–3 points **G2**: 4–6 points **G3**: 7 points	[24]
Elseragy et al., 2019 (Revised Grading)	TB WHO Grading	**RG1**: G1 and TB = 0/×20 **RG2**: G2 and/or TB ≤ 4/×20 **RG3**: G3 and/or TB ≥ 5/×20	[68]
Yu et al., 2019 (iBD Model)	GM Score BD Score	**iBD0**: BD0–1 and strong inflammatory reaction **iBD1**: BD1 and weak inflammation or BD2 and high inflammation **iBD2**: BD2 and weak inflammation	[67]
Duorato et al., 2020	TB TSR	**Low risk-tumor**: TB < 5/×200 and TSR < 50% **Intermediate risk-tumor**: TB < 5/×200 and TSR ≥ 50% or TB ≥ 5/×200 and TSR < 50% **High risk-tumor**: TB ≥ 5/×200 and TSR ≥ 50%	[16]
Sung et al., 2020	TSR TB/tumor area TIL/tumor area CD3/stromal area CD8/stromal area CD8/CD3	**Low risk group**: 0–3 points **Intermediate risk group**: 4–5 points **High risk group**: 6 points	[20]
Hori et al., 2021	Tumor CD163+ Bd score	**Group 1**: low-density subtype and Bd1 **Group 2**: high-density subtype or Bd2/Bd3 **Group 3**: high-density subtype and Bd2/Bd3	[36]

TB: Tumor Budding; DOI: Depth of Invasion; TSR: Tumor Stroma Ratio; GM: Glasgow Microenvironment; BA: Budding Activity; CNS: Cell Nest Size; TIL: Tumor Infiltrated Lymphocytes; WHO: Word Health Organization.

**Table 3:** Systematic review and meta-analysis regarding the prognostic role of Tumor Budding in Oral Squamous Cell Carcinoma.

Authors (Year)	Results	HR (95% CI)
Elseragy et al. (2022) [41]	High TB is significantly associated with poor OS and DSS in OTSCC. Significant heterogeneity between studies for OS.	OS: 2.32 (1.40–3.84) DSS: 1.89 (1.13–3.15)
Da Silva Dolens et al. (2021) [78]	High TB is significantly associated with poor OS, DSS, and DFS in OSCC. Significant heterogeneity between studies for OS.	OS: 2.96 (1.36–6.45) DSS: 1.72 (1.35–2.18)
Karjol et al. (2020) [35]	High TB is significantly associated with LNM and poor OS in OTSCC.	LNM: 3.07 (2.08–4.52) OS: 2.40 (1.84–3.14)
Wahab et al. (2020) [22]	The BD model is a prognostic factor of LNM in OSCC.	LNM: 2.02 (1.44–2.85)
Almangush et al. (2018) [42]	High TB is significantly associated with LNM, OS, and DFS in OSCC.	LNM: 7.08 (1.75–28.73) OS: 1.88 (1.25–2.82) DFS: 1.83 (1.34–2.50)
Almangush et al. (2018) [75]	TB evaluated on diagnostic incisional biopsies of OSCC is significantly associated with LNM.	NA
Zhu et al. (2018) [27]	High TB is significantly associated with poor OS in early-OSCC.	OS: 1.94 (1.30–2.89)

TB: tumor budding; OS: overal survival; LNM: lymph node metastasis; DFS: disease free survival; OSCC: oral squamous cell carcinoma; OTSCC: oral tongue squamous cell carcinoma; HR: Hazard ratio; CI: Confidence Interval; NA: not available.
